# A multilevel Bayesian approach to climate-fueled migration and conflict

**DOI:** 10.1038/s41598-025-25332-6

**Published:** 2025-11-21

**Authors:** Claire Palandri, Paulina Concha Larrauri, Andrew Gelman, Michael J. Puma, Upmanu Lall

**Affiliations:** 1 Harris School of Public Policy, University of Chicago, Chicago, IL USA; 2 Department of Earth and Environmental Engineering & Columbia Water Center, Columbia University, New York, USA; 3 Department of Statistics & Department of Political Science, Columbia University, New York, NY USA; 4 Columbia Climate School, Columbia University, New York, USA; 5 School of Complex Adaptive Systems & Water Institute, Arizona State University, Tempe, AZ USA

**Keywords:** Climate, Conflict, Migration, Multilevel model, Bayesian inference, Causal inference, Climate sciences, Mathematics and computing

## Abstract

Do climate conditions and extreme events fuel conflict and migration? This question has been widely studied using causal designs that exploit natural variation in climate variables, often analyzed with linear fixed-effects models. Yet in this setting, nonlinear relationships, distributional features of outcomes, and spatial heterogeneity can cause these models to violate core assumptions and yield unreliable inferences. We propose a multilevel Bayesian framework that accommodates such features while retaining identification strategies from natural experiments. We illustrate its potential with a representative analysis from the literature of the effect of temperature anomalies on conflict in Somalia. When outcome distributions suited to event counts are combined with partial pooling across regions, the apparent aggregate climate effect disappears and marked regional heterogeneity emerges, with positive associations in only a few southern regions and negative or uncertain effects elsewhere. Extending pooling across time further improves predictive ability. More broadly, the multilevel Bayesian framework offers a general strategy for strengthening both explanatory and predictive inferences about climate and social outcomes, supporting internal and external validity while efficiently accommodating heterogeneity even with small samples. This methodological bridge between econometric identification strategies and statistical modeling provides a robust foundation for interdisciplinary climate-conflict-migration research.

**Supplementary Information:**

The online version contains supplementary material available at 10.1038/s41598-025-25332-6.

## Introduction

Do climate conditions, and notably climate extremes, fuel or lead to conflict and migration? This question has been addressed by a rapidly growing number of empirical studies over the past two decades, with contributions from a variety of disciplines. In parallel, a large number of systematic reviews have followed, assessing both the diversity of methods used to analyze the statistical relationship between climate and social instability and whether a consensus emerges (see Supplementary Information S.1 online for an overview of reviews published in the past dozen years). Taken together, these reviews find mixed evidence of substantial climate impacts, largely reflecting the variability of specifications and data—such as how the onset of civil war is defined, or which types of displacement are considered^1–8^. The most recent literature reviews and meta-analyses continue to highlight a lack of consensus^9,10^, underscoring the persistent methodological and empirical challenges in establishing robust causal relationships. However, those studies within the new climate-economy literature^11^, which uses reduced-form models exploiting variation in climate variables to identify causal effects, tend to suggest a net effect of climate on conflict^12–14^.

Research designs that leverage such natural or quasi-experiments are positioned to credibly identify causal effects. Here, *quasi-experiments* refer to observational studies in which quasi-randomization of a variable occurs without researcher intervention, approximating the exogeneity of a randomized experiment, though without full random assignment. Applying such designs to the climate-migration-conflict nexus is of increasing relevance as climate conditions that were historically rare become more frequent and as adaptation becomes a more salient concern. However, this nexus departs substantially from the statistical setting that motivates the widespread linear reduced-form model, in at least two important ways. First, multiple features of the data-generating processes in this context diverge, more strongly than in other contexts, from the assumptions of the linear model. As a result, the best linear approximation to the conditional expectation function—what the linear reduced-form model provides—may not produce the desired information. Second, while causal inference and prediction can certainly be valuable on their own, we argue that causality questions in this area are often ultimately motivated by prediction; the causal effects of climate on conflict and migration are of increasing practical interest due to expected global changes in the distribution of climate. In effect, causality studies often make implicit or explicit prediction statements, based on their estimates of slope coefficients for climate regressors. Yet with many fixed effects and typically low explained variance, the uncertainty around such predictive statements may be rather large. This motivates turning to approaches that evaluate predictive performance explicitly, while accommodating and estimating heterogeneity in climate effects to support both explanation and prediction. The climate-migration-conflict nexus thereby prompts the use of statistical approaches that integrate both causal identification and predictive validity to provide meaningful insights. While traditional reduced-form linear models with fixed effects remain the standard approach in the causal inference literature, their limitations in capturing nonlinear climate responses and spatial heterogeneity efficiently limit explanatory power and policy relevance.

This paper proposes a multilevel Bayesian framework to analyze climate-fueled social instability. By explicitly modeling heterogeneous regional effects and nonlinear responses while leveraging quasi-experimental identification strategies, this framework offers a way of addressing the aforementioned limitations. We apply it to a dataset representative of the literature and directly compare its results with that from a linear fixed-effects approach. The application illustrates how the latter approach can not only obscure important heterogeneity in climate responses, but also mislead about the aggregate effect. In contrast, the multilevel Bayesian framework improves both the internal validity of causal estimates and out-of-sample predictive ability. In our application to the effect of temperature extremes on the number of conflict events in Somalia, we find that the apparent aggregate climate effect disappears once an outcome distribution suited to event counts is specified and regional pooling is introduced. Only a few southern regions drive positive estimates, whereas others show negative or highly uncertain effects. Moreover, regional partial pooling and then further temporal pooling incrementally improve predictive ability.

The remainder of the paper proceeds as follows. We first describe how specific features of climate-conflict data violate key assumptions of fixed-effects linear models, affecting both causal inference and predictive performance, and why these complementary statistical goals should be jointly considered. Next, we introduce the general multilevel Bayesian framework and describe a simple model for longitudinal data. We then illustrate its potential with the Somalia application and compare the results to those from the fixed-effects linear model. We finally discuss the broader implications of our findings for environmental social science research.

## Assumptions and constraints of fixed-effects linear models

Research designs focused on quasi-experiments with adjustments for confounders commonly use a reduced-form linear model to identify an average treatment effect. With longitudinal data, variation in climate variables is leveraged using a multivariate linear regression model, which includes indicator variables or ‘fixed effects’ for the units—e.g., locations—in the sample, as well as temporal fixed effects, to estimate the average effects of climate variables across units and periods. The multivariate linear regression model with fixed effects (MLR-FE) has the following general form:

1 $$\begin{aligned} y_{it} = \textrm{W}'_{it} \beta + \textrm{X}_{it}' \delta + \phi _i + \psi _t + e_{it}, \ \ \ \ e_{it} \overset{\text {iid}}{\sim }\text{ normal }(0,\sigma ); \ \ \ \ i = 1, \dots , n, \ \ t = 1, \dots , T \end{aligned}$$

where $y_{it}$ is the outcome variable for unit *i* at time *t*, $\textrm{W}_{it}$ is a vector of climate variables considered as treatment (often linear, sometimes including polynomials), $\textrm{X}_{it}$ is an optional vector of adjustment variables, $\phi _i$ is a vector of unit fixed effects, and $\psi _t$ is an optional vector of temporal fixed effects. The vector of key parameters of interest $\beta$ is estimated either by maximum likelihood, with the explicit assumption of normally-distributed errors $e_{it}$, or by least squares—where the same assumption is implicit for the typical tests of statistical significance. The motivation for using spatial and temporal fixed effects is to adjust for time-invariant and space-invariant unobserved confounders, respectively.

Assuming a strong causal identification strategy, the estimators of the slope parameters are unbiased conditional on the ability to adjust for all confounders. However, the validity, individual interpretation, and assessment of significance of the estimates $\hat{\beta }$ still rely on the modeling assumptions of additivity, linearity, spherical errors, normally-distributed errors, and non-collinear regressors^15^. No assumption is ever expected to be met perfectly with real-world data, and exact compliance with all these assumptions is not required for consistency. But in the climate-migration-conflict nexus in particular, the features of the data-generating process often suggest particularly large departures from the assumptions of the MLR-FE model which can become consequential for inference, and include:

- *Nonlinear functional forms.* Migration or conflict outcomes often have nonlinear relationships to environmental conditions, with frequent threshold effects^16^. In that context, the simple linear approximation to the conditional expectation function, even if accurate in capturing an average relationship, would fail to capture nonlinearities and thereby severely limit, if not mislead, the information carried by the slope coefficients, and have weaker predictive ability than a model that captures this behavior.
- *Limited outcome data.* In large samples, OLS estimators remain consistent under non-normal errors. But in this field, outcome data are measures of migration or conflict whose ranges of possible values are often limited—e.g., as they take the form of counts of rare events—producing strongly non-normal conditional distributions. Under such conditions, and especially with relatively small sample sizes, the reliability of conventional significance tests is diminished. This motivates outcome distributions better suited to the nature of the data, and systematic use of diagnostics. Short records across a large number of spatial units also raise the risk of high-influence observations, given the presence of extremes of the often right-skewed climate variables, or of the highly-skewed outcome variables.
- *Correlated climate regressors.* The regression on several climate variables—such as temperature, precipitation, and indices derived from them—limits the simultaneous interpretation of coefficients as individual effects when these variables are highly correlated. In such cases, only their joint effect can be interpreted reliably, while slope estimates risk instability. The literature notes concerns about multicollinearity; some studies address it for example by computing pairwise correlations among climate variables^17^ or by explicitly cautioning against interpreting coefficients simultaneously^18^.
- *Dependence structures and heterogeneous treatment effects.* Socio-ecological data in the climate-migration-conflict nexus often have a specific dependence structure—whether temporal, spatial or administrative^5^. Longitudinal data have a hierarchical structure, where a lower level is the repeated measure within the group across time and is nested within a higher level which represents the group-level data. So-called “sandwich” estimators, such as those proposed by Conley^19^ and Newey-West^20^, are a common way to adjust otherwise misleading estimates of parameter uncertainty for spatial and temporal autocorrelation. However, such adjustment is only valid to the extent that the dependence structure is correctly specified and the sample is sufficiently large. The MLR-FE model also typically fits a separate intercept per group and a homogeneous treatment effect, thereby modeling independent baseline outcome levels across groups, while assuming away similar group-level heterogeneity in the effect of the regressors. This *no-pooling* of intercepts and *full-pooling* of slopes corresponds not to absent or conservative assumptions, but instead to specific restrictive assumptions on the role of hierarchical structures in the data-generating process.

## Predictive ability strengthens causal inference

Research questions on the statistical relationships between climate and social outcomes that go beyond merely describing associations can often be classified as belonging to one of three categories:

1. Forward causal inference or “what if” questions, which seek to uncover the effects of causes^21^, e.g., *‘What is the average effect of heat waves on migration and conflict?’*
2. Reverse causal inference or “why” questions, regarding causes of effects, e.g., *‘What are the causes of the increased rates of interpersonal conflict observed in location* *X* *in the last few decades?’*
3. Prediction, e.g., *‘How many annual international migrants from region* *A* *to region* *B* *are expected by mid-century under a global average temperature increase of* $2^{\circ }\hbox {C}$ *above pre-industrial levels?’*

In the heterogeneous body of work concerned with capturing how changes in climate impact conflict and migration, the distinction between these three proximate goals and the focus on different assumptions between disciplines partly explain the variety of statistical approaches. In particular, the divide between explanatory and predictive goals has consequences at every step of the modeling process, from data preparation to the choice of regressors and model selection, while some steps may even virtually disappear, such as model evaluation in causal inference settings^22^.

In the climate-economy literature, studies generally state the goal of forward causal inference. At the same time, the motivation for prediction is often present—even if implicit—in estimating climate impacts. This is the case in causal inference studies in general: Rubin^23^—the seminal paper presenting the potential outcomes framework, on which typical causal inference approaches are based—highlights how the results of a (true or quasi) experiment are generally of interest only to the extent that the observed data are representative of a population of future treatment assignments, i.e., that the causal relationship has a certain predictive ability. This is especially relevant in the case of climate impacts. The answer to *“Do extreme climate events lead to conflict or migration?”* has become an increasing concern under the assumption that it tells us something about *“Will future climate changes bring more conflict or migration?”*.

This underlying motivation is revealed by many causal inference studies themselves, which include predictive statements or pair the estimates from explanatory causal models with climate model output to form projections. In the latter case, Carleton et al.^13^ aptly describe the need to account for the multiple sources of uncertainty, namely (i) the statistical uncertainty from the fitted model, (ii) the variation in climate model predictions, and (iii) the potential adaptation of societies to climate change that could alter the response function. We emphasize the concern that the original fitted model might have little predictive power if it uses only climate regressors and fixed effects while imposing strong assumptions on unmodeled group-level effects.

In analyses concerned with estimating causally-interpretable parameters, considering the predictive ability of the model would not only support the external validity of the analysis as well as an ultimate motivation of the research, but also bolster its internal validity. This has been notably illustrated in the study of conflict^24^. Prediction accuracy supports causal inference by providing an additional check on its assumptions, namely: the statistical assumptions about the data-generating process, and the aforementioned assumption of *subjective random sampling* of trials. Modeling assumptions about the data-generating process can be supported in the pre-modeling phase by using prior theory to dictate the model, but also post-modeling, by testing the model against reality, i.e., by assessing its predictive accuracy^25^. As social science knowledge can often be too limited for deriving precise specifications, prediction provides a way to evaluate whether these assumptions hold in practice.

In summary, in the climate-migration-conflict nexus, the features of the data-generating process and the importance of predictive interpretations motivate both a systematic examination of the fixed-effects linear model assumptions and the exploration of more flexible approaches. In the next sections, we explore leveraging the same quasi-experimental variation in climate through a framework that addresses these complementary statistical goals. We first introduce the implementation of the multilevel Bayesian framework with such causal identification strategies. We describe how it accommodates various conditional distributions of outcomes, models dependence in residuals and heterogeneous effects across spatial units, and propagates uncertainty into projections under simulated climate conditions.

## The multilevel Bayesian framework

This section introduces the multilevel Bayesian framework as a generalization of the fixed-effects model. We first restate the fixed-effects specification, recasting it within this framework to highlight its underlying assumptions, and then show how a multilevel structure with partial pooling relaxes them.

Longitudinal data have a hierarchical structure, where repeated measures at the lower level (time within group) are nested within higher-level group data, for example, regions. Recast in a hierarchical (i.e., multilevel) modeling framework, the MLR-FE model (1) fit to such data embeds restrictive assumptions about between-group variation: regional intercepts are completely independent or *unpooled,* while climate variables have homogeneous or *fully-pooled* effects across regions. This *varying-intercepts fixed-slopes* model is represented in Eq. (2), where the conditional distribution of the outcome variable is generalized as $\mathscr {F}(\mu , \theta )$ with mean $\mu$ and other parameters $\theta$, and we consider the example of a vector of two climate-related predictors, such that $\beta = (\alpha , \gamma )'$ and $\textrm{W}_{it} = (A_{it}, B_{it})'$:

2 $$\begin{aligned} y_{it} \sim \mathscr {F}(\mu _{it}, \theta ), \ \ \mu _{it} = \alpha A_{it} + \gamma B_{it} + \textrm{X}_{it}' \delta + \phi _i + \psi _t; \ \ \ i = 1, \dots , n; \ \ t = 1, \dots , T. \end{aligned}$$

In this fixed-effects model, each regional intercept $\phi _i$ is estimated from the data of the given region *i* only, which is equivalent to assuming that the intercepts belong to a joint distribution with an infinite variance. Homogeneous slope parameters, on the other hand, implicitly represent the other extreme of zero variance between regions. It is often reasonable to assume instead some degree of closeness between effects across regions, and hence use a compromise between *full pooling* and *no pooling* of regional coefficients. In effect, one may assume that they belong to a common distribution and let the degree of pooling be determined by the data. This is easily implemented with a multilevel model, where within-group variation is explicitly modeled at the lower level, and between-group variation at the higher level. Modifying model (2) to allow for such *partial pooling* of both slopes and intercepts across regions results in the two-level model (3), where the vector of region-level coefficients is assumed to follow a joint multivariate normal (MVN) distribution. Group averages of the causal variables are added as covariates to address concerns of bias from time-invariant confounders introduced by the modeled region-level effects. This model is sometimes referred to as the “within-between random effects” model^26^ or the “correlated random effects” model^27^. By including the group averages as regressors, the problematic correlation between the treatment variables and the group effects is removed from the group-level error term, resolving the concern of bias from group-level (here: time-invariant) confounders in a manner similar to the fixed-effects model^28^.

3 $$\begin{aligned} \begin{aligned}&y_{it} \sim \mathscr {F}(\mu _{it}, \theta ), \ \ \mu _{it} = a_i A_{it} + b_i B_{it} + \textrm{X}_{it}' \delta + f_i + \eta _1 \bar{A}_i + \eta _2 \bar{B}_i + \psi _t; \ \ \ i = 1, \dots , n; \ \ t = 1, \dots , T \\&\hspace{-0.2cm} \begin{bmatrix} a_i \\ b_i \\ f_i \end{bmatrix} \sim \text{ MVN }\left( \begin{bmatrix} \alpha _0 \\ \gamma _0 \\ \phi _0 \end{bmatrix}, \begin{bmatrix} \sigma ^2_a & \sigma _{ab} & \sigma _{af} \\ \sigma _{ab} & \sigma ^2_b & \sigma _{bf} \\ \sigma _{af} & \sigma _{bf} & \sigma ^2_f \end{bmatrix} \right) \end{aligned} \end{aligned}$$

In addition to relaxing the constraints on the distribution of regional coefficients, the multilevel structure allows them also to be informed by group-level predictors. In this simplest form of the model, the serial or spatial dependence structure is not explicitly represented; however, it could be modeled by including relevant additional regressors—such as lags of the dependent variable—alongside $\textrm{X}_{it}$.

This model can be estimated in a frequentist or Bayesian framework. We emphasize the Bayesian framework because it provides full posterior distributions for group-level and population parameters, regularizes estimation in small samples, and facilitates evaluation of predictive performance through posterior predictive checks. Specifically in the climate-conflict-migration context, it simultaneously allows for: (i) accounting for the typically limited nature of migration and conflict outcome data through an adequate choice of $\mathscr {F}()$, (ii) partially pooling the intercept and slope coefficients across groups efficiently, and (iii) propagating uncertainty in parameter estimation. The posterior distributions produced can indeed be combined with simulations from climate models to account for the uncertainty in estimation when projecting outcomes.

The same identification strategies based on quasi-experiments and adjustments for confounders can be leveraged within this framework, which generalizes the typical linear model. Like any statistical approach, the multilevel Bayesian model also rests on assumptions: the functional form of the model, the specification of the outcome distribution, and the priors assigned to parameters all shape the resulting inferences. It is therefore essential to question these assumptions, and use tools such as posterior predictive checks to assess their plausibility. This framework also implies tradeoffs. The most prominent are higher computational costs and a potential reduction in the effective degrees of freedom for statistical tests. A further, though presumably less consequential, tradeoff is that of one linear assumption for another. Indeed, with a *non-identity* link function, the bias from time-invariant confounders is fully removed by including the group average of the causal variable as a covariate only if the random effect is a linear function of this average. That being said, simulations suggest that the remaining bias remains small in most situations^26^, and additional functions of the group average can also be included to characterize more flexible functional forms of the correlation. Although these tradeoffs exist, we propose that when model diagnostics show strong departures from the assumptions of the typical linear model—such that *t* tests of the coefficients of the best linear approximation to the conditional expectation function do not provide the desired information—and when heterogeneity in treatment effects or prediction are of interest, as is generally the case in the climate-economy literature, these tradeoffs may be worthwhile.

Multilevel models have been used to study the relationship between climate and migration. For example, Nawrotzki et al.^29^ use a two-level regression model to account for the hierarchical structure of their data (households nested in municipalities). In a similar setting, Nawrotzki et al.^30^ consider a third level and include state-level predictors, explicitly addressing the fact that “migration decisions are influenced by forces operating at different scales.” In both studies, the central findings are the variation of the climate-migration association by location characteristics. However, only intercepts are modeled as random effects; slopes are fully pooled across units. The estimated relationships are not causally interpretable because they lack a strong identification strategy, and the predictive ability of the model is also not assessed. In the context of conflict outcomes, Burke et al.^12^ use a hierarchical Bayesian model to conduct a meta-analysis of estimates from the literature, themselves selected for their use of the MLR-FE framework. In the present paper, we suggest instead using the multilevel Bayesian framework in combination with established identification strategies, to combine the focus on identification with the consideration of modeling assumptions and account for heterogeneity by modeling slope parameters in addition to intercepts. This framework can be adopted not solely in settings with hierarchical levels within the spatial dimension, but in any longitudinal data.

In the next section, we apply this model to a dataset representative of the literature and compare the insights obtained with those from the fixed-effects linear model.

## Application: temperature anomalies and civil war in Somalia

We consider the dataset on climate and conflict in Somalia used in Maystadt and Ecker^31^, hereafter M&E. This choice is motivated by the availability and representativeness of these data: their analysis was conducted within the MLR-FE modeling framework, and has been cited in a substantial number of literature reviews published since its original publication^3,4,6,10,12–14,32^.

**Table 1:** Main variables in M&E’s original reduced-form specification.

Description	Name	Original resolution and processing steps	Source
Count of violent conflict events	*conflict*	Region × month	ACLED (2011)^35^
Temperature anomaly	*TA*	0.5° × 0.5° grid × month (average of daily maximum Ts) a. T interpolated to region centers by kriging; b. anomaly computed w.r.t. 1980–2009; c. anomaly averaged over 3 months	CRU TS 3.1 (2008)^36^
Drought length	*DL*	count of consecutive months with positive *TA* values	CRU TS 3.1 (2008)^36^
Precipitation anomaly	*PA*	0.5° × 0.5° grid × month (total precipitation P) a. P interpolated to region centers by kriging; b. anomaly computed w.r.t. 1983–2009; c. anomaly averaged over 3 months	CRU TS 3.1 (2008)^36^

**Table 2:** Frequentist estimates of slope coefficients of the single-level models.

Conditional distribution	Normal (original^a^ SEs)	Normal (corrected SEs)	NB	NB
*TA*	0.71	0.71	0.01	0.08
	(0.25)	(0.37)	(0.12)	(0.11)
*DL*	0.08	0.08	0.02	0.02
	(0.01)	(0.04)	(0.01)	(0.01)
*PA*	$-0.47$	$-0.47$	$-0.08$	$-0.07$
	(0.20)	(0.31)	(0.16)	(0.15)
Region FEs	$\checkmark$	$\checkmark$	$\checkmark$	$\checkmark$
Year-month FEs	$\checkmark$	$\checkmark$	$\checkmark$	$\checkmark$
Region-month FEs	$\checkmark$	$\checkmark$	$\checkmark$	
*N*	2808	2808	2808	2808
Multiple R^2^		0.43		
Within^b^ R^2^	0.17	0.17		
Adjusted within R^2^		0.04		
AIC		16121	5837	5639

The raw regression coefficients are displayed; they represent additive changes in *y* in Gaussian models, and additive changes in $\log (y)$ in negative binomial models, where *y* is the number of conflicts.

^a^ The ‘original’ standard errors (SEs) are those reported in M&E. They were computed using a version of the ols_spatial_HAC function by Hsiang^37^ which miscalculated the weights for serial autocorrelation. Using the corrected version (v3) in the authors’ Stata code results in the ‘corrected’ standard errors in the adjacent column.

^b^ The within or ‘projected’ $R^2$ corresponds to the $R^2$ of the mean-deviated regression, i.e., after removing region fixed effects, and represents the share of the variation in the outcome within regions that is captured by the model.

The hypothesis originally tested with this dataset is that temperature extremes are an indirect determinant of conflicts in Somalia operating primarily through the channel of livestock prices. The dataset contains longitudinal, monthly data at the scale of administrative regions, over the period 1997–2009. Supplementary Figure S4 online shows a map of these 18 regions. The causal identification strategy uses the exogeneity of two main explanatory variables capturing the level and duration of temperature extremes—‘temperature anomaly’ *TA* and ‘drought length’ *DL*—and controls for ‘precipitation anomaly’ *PA*. The outcome of interest is the number of violent conflict events. The main variables are listed in Table 1 along with the raw data sources and transformation steps. The dataset also contains livestock prices, instrumented by the climate variables in a two-stage least-squares fixed-effect model to explore the mechanism driving the reduced-form relationship. As that instrumental variable specification adopts the same functional form and treatment of the error terms as the reduced-form—and our study focuses on these modeling assumptions, not causal-identification assumptions—we present here only the reduced-form model.

### Inferences from the MLR-FE model and other single-level specifications

We first estimate the effects of the climate variables of interest using the MLR-FE model, presented in Eq. (4), where *i* refers to the region, *m* the calendar month, *y* the year, and $e_{imy}$ represents the error term.

4 $$\begin{aligned} \begin{aligned}&\textit{conflict}_{imy} = \alpha TA_{imy}+ \gamma DL_{imy} + \delta PA_{imy} + \phi _i + \psi _{my} + \omega _{im} + e_{imy}, \ \ \ e_{imy} \sim \text{ normal }(0,\sigma ); \\&\hspace{1cm} i = 1, \dots , 18; \ \ m = 1, \dots , 12; \ \ y = 1, \dots , 13 \end{aligned} \end{aligned}$$

**Fig. 1:**
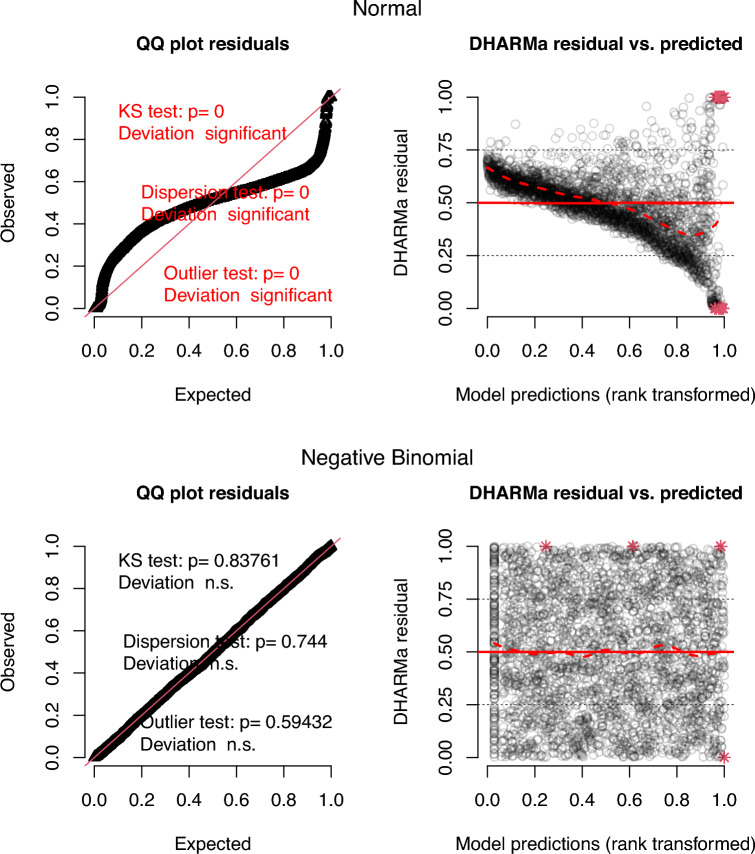
Residual plots of the reduced-form model assuming a conditional normal distribution (top) or negative binomial distribution (bottom). Left: quantile-quantile plot of the residuals, including Kolmogorov-Smirnov (KS) test for the goodness of fit of the residuals to the specified distribution. Right: standardized residuals against model predictions; red stars represent outliers.

Like M&E, we include 18 region fixed effects captured by $\phi _i$, 156 month-year fixed effects $\psi _{my}$, and 216 region-month fixed effects $\omega _{im}$. Temporal and spatial dependencies are accounted for simultaneously by estimating the variance-covariance matrix of the error term following the methods of Newey and West^20^ and Conley^19^ with uniform weighting kernels. Spatial dependency is assumed to disappear beyond a cutoff point of 263 kilometers, corresponding to the maximum distance between the centroids of any pair of neighboring regions, and time dependency is allowed up to four months. The summary of the results is presented in the first two columns of Table 2, and diagnostic plots of the residuals are presented in the first row of Fig. 1. Additional diagnostic plots are provided in detail in the Supplementary Information S.2 online.

The normal quantile-quantile diagnostic plot shows a strong departure from normality in the distribution of the residuals. A Shapiro-Wilk test and a Kolmogorov-Smirnov test further confirm this departure. *TA* and *DL* have a correlation coefficient of 0.28 when fixed effects are removed (see Supplementary Fig. S1 online). The computation of the correlations between explanatory variables by region shows that *PA* and *DL* are highly correlated in the northern regions of Awdal, Nugaal, Sool, and Togdheer. The share of the variation in outcomes within regions that is explained by the model, captured by the *within R*^2^, is negligible when adjusted for the degrees of freedom. The correlations of climate variables, the non-normality of the residuals, and the lack of information on the true correlation structure of the residuals, render the *p*-values of the regression coefficients unreliable indicators for causal identification.

**Fig. 2:**
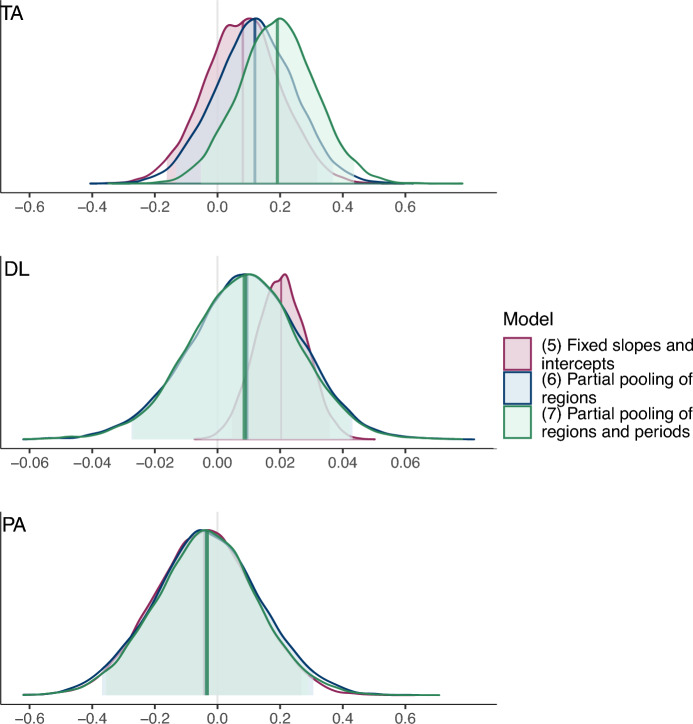
Posterior distributions of the central slope coefficients of the Bayesian negative binomial models. Pink: single-level model (varying-intercept, fixed-slope), parameters $\alpha , \gamma , \delta$. Blue and green: multilevel models (blue: intercepts and slopes partially pooled across regions; green: intercepts partially pooled across regions and periods, slopes partially pooled across regions), hyperparameters $\alpha _0, \gamma _0, \delta _0$. 95% credible intervals are shown as shaded areas under the curves around the median point estimates.

We therefore explore the robustness of the results to an alternative model specification better suited to the data-generating process. We consider a negative binomial (NB) conditional distribution to account for the count nature of the outcome data, its positive skew and the frequency of zero values:

5 $$\begin{aligned} \begin{aligned}&\textit{conflict}_{imy} \sim \text{ NB }(\mu _{imy}, \Theta ); \ \ \ i = 1, \dots , 18; \ m = 1, \dots , 12; \ y = 1, \dots , 13 \\&\hspace{1cm}\mu _{imy} = \alpha TA_{imy} + \gamma DL_{imy} + \delta PA_{imy} + \phi _i + \psi _{my} \end{aligned} \end{aligned}$$

The second row of Fig. 1 presents the diagnostic plots of this NB model (5), estimated as a generalized linear model with logarithmic link function. As expected, we no longer observe a stark departure from the modeling assumptions; the Akaike information criterion (AIC) is also substantially lower. With this distribution, we find that the key climate variables of interest are not statistically significant (Table 2).

### A multilevel Bayesian model of climate-fueled conflict

We now relax the assumptions of no-pooling of baseline effects and full pooling of climate effects across regions by generalizing to the multilevel negative binomial model (6), which partially pools slopes and intercepts across regions, and we estimate it in the Bayesian framework. Removing the region-month interaction term $\omega _{im}$ present in the single-level model specifications gives comparable results and is more computationally efficient, so we omit it from the multilevel model for simplicity. All parameters, including the hyperparameters of the between-group covariance matrix, are assigned *weakly informative* priors, i.e., prior distributions that mildly constrain parameters toward plausible values to regularize estimation without overwhelming the information in the data (see the Supplementary Information S.4 online for details).

6 $$\begin{aligned} \begin{aligned}&\textit{conflict}_{imy} \sim \text{ NB }(\mu _{imy}, \Theta ); \ \ \ i = 1, \dots , 18; \ \ m = 1, \dots , 12; \ \ y = 1, \dots , 13\\&\hspace{1cm}\mu _{imy} = a_i TA_{imy} + b_i DL_{imy} + c_i PA_{imy} + f_i + \eta _1 \overline{TA}_i + \eta _2 \overline{DL}_i + \eta _3 \overline{PA}_i + \psi _{my} \\&\begin{bmatrix} a_i \\ b_i \\ c_i \\ f_i \end{bmatrix} \sim \text{ MVN }\left( \begin{bmatrix} \alpha _0 \\ \gamma _0 \\ \delta _0 \\ \phi _0 \end{bmatrix}, \begin{bmatrix} \sigma ^2_a & \sigma _{ab} & \sigma _{ac} & \sigma _{af} \\ \sigma _{ab} & \sigma ^2_b & \sigma _{bc} & \sigma _{bf} \\ \sigma _{ac} & \sigma _{bc} & \sigma ^2_c & \sigma _{cf} \\ \sigma _{af} & \sigma _{bf} & \sigma _{cf} & \sigma ^2_f \end{bmatrix} \right) \end{aligned} \end{aligned}$$

Figure 2 shows the posterior distributions of the central slope coefficients of the two negative binomial models—$(\alpha , \gamma , \delta )$ for the single-level model (5) and $(\alpha _0, \gamma _0, \delta _0)$ for the multilevel model (6)—estimated using a Bayesian framework. For each parameter of interest, Bayesian estimation produces a full posterior distribution, which represents the updated uncertainty about the value of the parameter after observing the data. To compare this estimation to results from a frequentist approach, like model (5) (which produces a single point estimate with an associated standard error and confidence intervals), we compute summary statistics of the posterior distribution, specifically the median as a measure of central tendency and 95% credible intervals. As expected, the median values of the distributions from the fixed-slope Bayesian model are virtually identical to the frequentist point estimates. However, in the partial pooling model, the distribution of the mean slope coefficient for *DL* shifts towards zero, making the evidence of an effect of the climate variable is inconclusive.

**Table 3:** Bayesian central estimates of slope coefficients of single-level and multilevel models.

Model Condit. distribution	(4) Normal	(5) NB	(6) NB	(7) NB
*TA*	0.71	0.08	0.12	0.19
	(0.30)	(0.12)	(0.13)	(0.12)
*DL*	0.08	0.02	0.01	0.01
	(0.02)	(0.01)	(0.02)	(0.02)
*PA*	$-0.47$	$-0.04$	$-0.03$	$-0.03$
	(0.31)	(0.16)	(0.17)	(0.16)
$\overline{TA}_i$			$-11.6$	$-11.13$
			(2.37)	(2.23)
$\overline{DL}_i$			0.37	0.36
			(0.19)	(0.18)
$\overline{PA}_i$			1.64	1.85
			(3.07)	(3.07)
$\overline{TA}_{my}$				0.57
				(0.29)
$\overline{DL}_{my}$				$-0.07$
				(0.04)
$\overline{C}_{my}$				0.01
				(0.00)
Region	FEs	FEs	Pooled	Pooled
Month	FEs	FEs	FEs	Pooled
*N*	2808	2808	2808	2808
ELPD	$-8090.2$	$-2861.7$	$-2833.8$	$-2809.8$
$\text {ELPD}_{\text {diff}}$	$-5280.3$	$-51.8$	$-24$	0
SE[$\text {ELPD}_{\text {diff}}$]	186.4	13.8	9.5	0

For each regressor, the table display summaries of its marginal posterior distribution: the distribution’s median (top sub-row) and an estimate of the distribution’s standard deviation (bottom sub-row, in parentheses) based on a scaling of the median absolute deviation around that median. $\text {ELPD}_{\text {diff}}$ corresponds to the difference in expected log predictive density (ELPD) between models, and SE [$\text {ELPD}_{\text {diff}}$] to the standard error of that difference, where the reference is the model with the largest ELPD (model (7)).

**Fig. 3:**
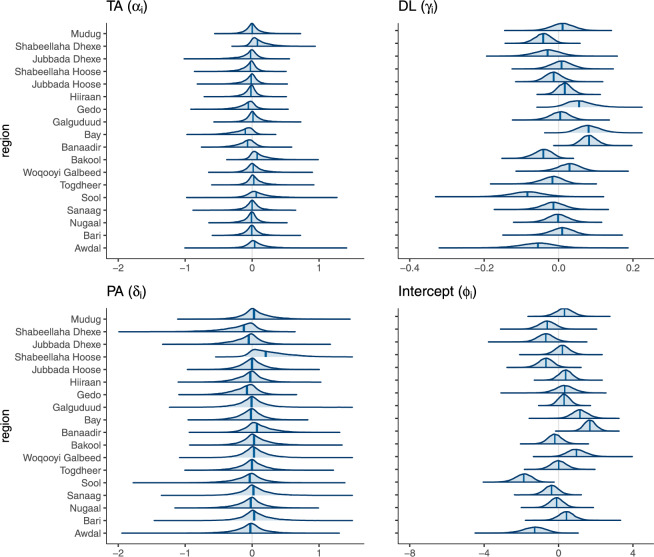
Posterior distributions of the partially-pooled region-specific intercepts and slope coefficients (model (6)). 95% credible intervals are shown as shaded areas under the curves around the median point estimates.

The posterior distributions of the partially-pooled slope coefficients $(a_i, b_i, c_i)$ provide some insight into what drives these higher-order effects. Figure 3 shows substantial heterogeneity in the effect of *DL* between regions, with only a few southern regions (Banaadir, Bay, and Gedo) actually driving the positive causal relationship, while others experience negative effects, which helps explain the relatively weak results in the full-pooling model. The Banaadir and Bay regions experienced the lowest numbers of months without any conflict throughout the study period. The uncertainty around the parameters also shows large differences between regions. In the northern regions of Awdal and Sool, the large uncertainty reflects the low total number of conflicts over the study period (one and four, respectively).

#### Predictive ability

 Considering the model’s predictive ability—whether for model evaluation or for simulation—is hindered by the presence of temporal fixed effects. Indeed, what are the values of the coefficients on year-month indicator variables for future periods? Similarly to pooling region intercepts in place of using region fixed effects, the assumption of unrelated effects across months can be relaxed by pooling them, i.e., by modeling them as random effects. We can then form predictions for a time period outside of the sample by sampling from their estimated distribution. We explore this in model (7), where we further add predictors for the country-wide period effect $w_t$. We include the group averages of the causal variables (taken over the regions within each period) to address unbiasedness concerns, as well as a time-varying climate forcing $C_t$ experienced across all regions, and model $w_t$ as a simple linear function of this predictor. Given a climate time series $c_{it}$, such as a drought index—or in our case the precipitation anomaly $PA_{it}$—we construct the climate forcing predictor $C_t \equiv \sum _i \frac{c_{it}}{\mathbb {Q}_{c_i}(.5)}$ which emphasizes threshold exceedance. Here $\mathbb {Q}_{c_i}(.5)$ is the median of the climate time series. The general motivation for including group-level predictors is that they may reduce unexplained group-level variation and thus yield more precise estimates than by shrinking all groups equally toward the population mean. For this specific predictor, the motivation is two-fold: we may want to assess whether conflict responds to a pervasive spatial impact or an aggregate climate anomaly aside from a regional climate anomaly, and we would expect it to be related to tail behavior of the same sign across locations. We also consider an alternative definition of the climate forcing that captures deviations from the average, with very similar results (see Supplementary Information S.3.2 online).

7 $$\begin{aligned} \begin{aligned}&\textit{conflict}_{imy} \sim \text{ NB }(\mu _{imy}, \Theta ),\ \ \ i = 1, \dots , 18; \ \ m = 1, \dots , 12; \ \ y = 1, \dots , 13; \\&\hspace{1cm}\mu _{imy} = a_i TA_{imy} + b_i DL_{imy} + c_i PA_{imy} + f_i + \eta _1 \overline{TA}_i + \eta _2 \overline{DL}_i + \eta _3 \overline{PA}_i + w_{my} \\&w_{my} \sim \text{ normal }(\psi _0 + \psi _1 \overline{TA}_{my} + \psi _2 \overline{DL}_{my} + \psi _3 C_{my}, \ \sigma _w) \\&\begin{bmatrix} a_i \\ b_i \\ c_i \\ f_i \end{bmatrix} \sim \text{ MVN }\left( \begin{bmatrix} \alpha _0 \\ \gamma _0 \\ \delta _0 \\ \phi _0 \end{bmatrix}, \begin{bmatrix} \sigma ^2_a & \sigma _{ab} & \sigma _{ac} & \sigma _{af} \\ \sigma _{ab} & \sigma ^2_b & \sigma _{bc} & \sigma _{bf} \\ \sigma _{ac} & \sigma _{bc} & \sigma ^2_c & \sigma _{cf} \\ \sigma _{af} & \sigma _{bf} & \sigma _{cf} & \sigma ^2_f \end{bmatrix} \right) \end{aligned} \end{aligned}$$

**Fig. 4:**
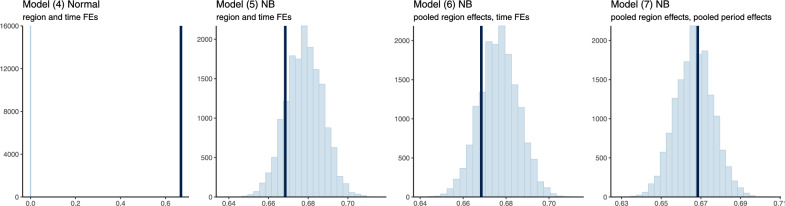
Graphical posterior predictive check: comparison of the proportion of zeros in observed vs simulated datasets. For each model, 8000 datasets are simulated from the posterior predictive distribution using the observed predictors. The light blue histogram represents the distribution of the value of the statistic (the proportion of zeros) across the simulated datasets. The dark blue vertical line is the value of this statistic for the observed sample.

Figure 4 shows a graphical comparison of relevant statistics of the observed data—namely, the proportion of zeros, due to the count nature of the outcome—against replicated datasets from the fits of the different models considered. This comparison of the posterior distributions shows that the original Gaussian model is not able to capture the count nature of the data. The negative binomial models with no pooling or partial pooling across regions provide better fits, and the partial pooling of temporal effects produces the best fit for the data. The higher performance of the multilevel models is also supported by cross-validation. We estimate the expected log predictive density (ELPD) of each model using leave-one-out cross-validation, then compute the differences between these ELPD estimates along with the standard errors of these differences. The results in Table 3 show the same hierarchy in expected predictive accuracy as in the comparison of posterior predictive statistics. We also replicate the partial-pooling model with the original Gaussian functional form, to assess how the predictive performance changes when using a multilevel model, compared to the original specification (see Supplementary Information S.3.1 online). We find, as captured by the ELPD, that the partial-pooling models perform marginally better than the no-pooling model; however, they do not match the performance of the NB model which accounts for the count nature of the outcome data.

The application of hierarchical Bayesian models to this dataset illustrates how the standard MLR-FE model can be readily recast in the hierarchical Bayesian framework, with a better suited conditional distribution for the response variable, inferences on parameters generated using simulations—which enable the propagation of uncertainty if estimates are later used in projections—and the partial pooling of regression coefficients, and can thereby allow one to analyze and model differences across groups efficiently. The posterior distributions obtained can easily be combined with simulations similar to those proposed in M&E, e.g., of increases in one standard deviation in the main climate variables, or of projected changes from global climate models under climate scenarios, to address what we can reasonably say about questions akin to *‘Will future climate changes bring more conflict or migration?’* and with how much uncertainty.

## Discussion

The hierarchical Bayesian approach to studying climate-fueled migration and conflict addresses several statistical principles of particular concern in this interdisciplinary field. First, prediction and identification of causal effects are complementary pursuits. Together, they support the modeling assumptions and thus the internal validity of the statistical analysis while strengthening its external validity, which is generally of practical interest. Reporting both the model’s explanatory power and its predictive power, and considering the latter for model selection, may strengthen inference from natural experiments^22^. Second, the linear approximation of the conditional expectation function of the outcome—which is the target of the linear reduced-form model—may be misleading when the data-generating process departs substantially from the modeling assumptions. Separately from the identifying assumptions supported by quasi-experimental variation and the ability to adjust for confounders, the validity of inferences also rests on the underlying assumptions of the estimated model. An assessment of the key assumptions such as the conditional distribution of the outcome, the absence of high influence observations, and the non-collinearity of regressors is hence of particular interest and may be provided alongside the estimation results—see for example Cohen et al.^33^. Third, the response to climate variables can vary across spatial units but with the ability to estimate spatial sensitivity limited by the sample size. In such cases, a fully pooled model for the slope coefficients, as is typical in the MLR-FE framework, represents a tradeoff between the efficiency gained by using a larger sample size (by pooling across all locations) and the potential bias at each unit. Instead, generalizing to a partial pooling approach combines information across units, shrinking uncertainty while reducing bias at individual spatial units. This multilevel structure provides a principled way to assess whether variation in climate sensitivity across groups is meaningful and what observed attributes it may depend on.

The multilevel Bayesian framework, which is a generalization of the linear reduced-form model, brings the analysis closer to addressing the three concerns above, thereby strengthening inference from quasi-experiments in the climate-migration-conflict nexus. The benefits of such models and inference methods have been shown abundantly in the statistics literature and are relevant in many settings using observational data to generate causal and predictive inferences. They come down to considering more general statistical frameworks to learn from quasi-experimental data, and selecting models that approximate the data-generating process; essentially applying the recommendation of “a combination of the economists’ focus on identification strategies and the statisticians’ ability to build more complicated models to assess what might happen if the strict assumptions fall apart”^34^. This study emphasizes and illustrates how these benefits are particularly salient for studying the relationship between climate and social instability. In this application, most of the contrast in estimated climate effects arises from adopting a negative binomial outcome distribution, which better reflects the count nature of the conflict data. The Bayesian framework complements this correction by enabling partial pooling and coherent assessment of uncertainty and predictive performance, extending the analysis to capture heterogeneity and strengthen inference within a unified structure. Our findings suggest that a multilevel Bayesian approach has the potential to support the internal validity, external validity, and efficiency of inferences, providing a robust foundation for understanding the impacts of changes in climate on social outcomes of interest. This methodological bridge between econometric identification strategies and complex statistical modeling capabilities addresses a critical need in interdisciplinary climate impact research, where collaboration across disciplinary boundaries is essential.

## Supplementary Information

Supplementary Information.

## Data Availability

The code and datasets generated and used for the study are available on Zenodo at https://zenodo.org/records/17165112.
