# Supplementary material for: A multilevel Bayesian approach to climate-fueled migration and conflict
Source: Sci Rep. 2025 Nov 21;15:41268. doi: 10.1038/s41598-025-25332-6 (PMC12639035; doi:10.1038/s41598-025-25332-6)
Supplement: Supplementary file 1 — Supplementary Information. [file 41598_2025_25332_MOESM1_ESM.pdf]

Supplementary Information

S.1 Overview of systematic reviews of the climate-migration-conflict literatures (2013-2024)

Over the past twelve years (2013-2024), the literature examining relationships between climate change, migration, and conflict has been subject to extensive systematic review, with at least 21 reviews published during this period—12 focusing on migration[1,2,5,9,38–45] and 9 on conflict[3,4,6,10,12–14,32,46]. This proliferation of meta-analytical work reflects both the growing importance of the topic and the persistent challenges in reaching consensus about climate impacts on human mobility and social stability.

The systematic reviews highlight a high methodological diversity. Early syntheses identified distinct modeling approaches including spatial vulnerability models, hazard analysis models, and agent-based modeling for migration studies[40,43]. The econometric literature has favored multivariate regression models and gravity models, while simulation approaches have employed agent-based models and integrated assessment models. The inference approaches used also differ. At the highest level, we can first separate two classes of models: (i) computational models, which use simulations to study systems of interest; these include agent-based models[47,48], system dynamics models[49], computable general equilibrium models[50], and integrated models[51]; (ii) empirical analyses using regression models, from simple least squares to multilevel generalized linear models and nonparametric predictive models. Regression-based approaches can be classified along multiple dimensions, such as the functional form of the relationship between predictor and outcome variables, the assumed conditional distribution of the outcome variable, and the stated end goal of the statistical exercise—notably prediction or causal inference (which can be considered as conditional prediction). Table S1 illustrates the diversity of approaches as they leverage some combination across these key dimensions, based on a sample of studies in the literature–each cited by at least one of the 21 literature reviews. Multiple reviews also emphasize that the lack of comparable data and standardized measures across contexts prevents making generalizations that would facilitate the development of future scenarios[5,40]. The literature does not use core concepts and variables in a standardized manner, with different operationalizations of dependent variables (onset versus incidence of conflict, various types of displacement) and independent variables requiring nuanced theoretical approaches[3].

The migration literature presents complex findings. Reviews consistently identify that environmental change alone does not cause migration; rather, it is the interaction of environmental factors with non-environmental factors that drives mobility decisions[5,39]. This insight has led to the development of frameworks distinguishing between vulnerability and capability effects, where climate shocks affect both household resources necessary for migration and the risk of remaining in place[41]. Several important patterns emerge: climate variability produces different responses than long-term climate change, slow-onset events produce different migration outcomes than fast-onset events, and the relationship between climate severity and migration is non-linear, with threshold effects determining whether vulnerability or capability channels dominate. The most recent and largest meta-analysis synthesizes 96 studies and finds an overall average effect on migration that is small for both slow- and rapid-onset events, but positive and significant[9]. It also highlights that across studies, climate is found to play diverse and even opposite roles: a driver of migration, a constraint to mobility, or having no significant role among other drivers—highlighting the fundamental heterogeneity in climate-migration relationships.

The conflict literature presents a more polarized landscape. Early systematic reviews found little consensus on the physical pathways connecting climate change to violent conflicts[3,52]. However, the emergence of the "climate-economy" literature, employing reduced-form econometric models with high-frequency climate variation and fixed effects, has claimed to establish robust causal relationships. Meta-analyses that examined studies meeting strict econometric criteria—requiring time-series variation, location-specific fixed effects, and trend controls—found consistent support for climate-conflict links[12,14]. However, reviews also note a frequency-identification trade-off: populations evolve faster than many low-frequency climatic changes of interest, undermining the comparability of control and treatment populations over time[12,13], and highlight the limited insights into mechanisms and policy pathways[6]. The latest review still notes "a remarkable inconsistency of evidence among publications"[10].

Across the climate-migration-conflict nexus, a critical finding across assessments is the presence of significant methodological limitations and selection biases. Methodological choices substantially influence the probability of finding climate effects, with different econometric techniques, climate measures, and outcome definitions producing systematically different results[1]. The literature's focus on econometric identification has excluded valuable predictive modeling approaches[1,41], while geographic coverage shows systematic biases with certain regions over-represented while other conflict-affected or climate-vulnerable regions remain understudied. The reviews consistently identify the absence of robust theoretical frameworks as a fundamental limitation. This theoretical gap contributes to the proliferation of empirical approaches without clear guidance on appropriate model specifications or expected effect magnitudes. A recurring concern is also the external validity of historical relationships for future climate scenarios, as adaptation could fundamentally alter observed relationships[13,32].

Conclusion. After more than a decade of systematic review, the persistence of mixed findings across multiple comprehensive reviews suggests that methodological diversity, while valuable, may be insufficient to resolve core empirical questions in the

climate-migration-conflict literature without stronger theoretical foundations and more attention to predictive performance alongside causal identification. This suggests that purely econometric approaches to causal identification may need to be complemented by more flexible modeling frameworks that can accommodate heterogeneity, non-linearity, and complex interactions while maintaining rigorous standards for causal inference. This integration of econometric identification strategies with predictive modeling—as advocated in our main analysis—represents a promising direction for advancing both scientific understanding and policy relevance in this critical area of research.

S.2 Diagnostic plots of the fixed effects linear model

Figure S1 presents scatterplots of the model's main variables—adjusted for the region, month-year and region-month fixed effects by using the residuals of a linear regression of each variable on these fixed effects. The results suggest no obvious strong relationships between the outcome and the explanatory variables.

Figure S2 presents diagnostic residual plots of the model. From left to right, top to bottom, the sub-panels provide the following information:

- "Residuals vs. Fitted" shows whether the residuals are equally spread around a horizontal line or display a pattern—suggesting a nonlinear relationship that was not captured by the model.
- "Normal Q-Q" shows how the distribution of the residuals aligns with a normal distribution.
- "Scale-Location" presents the spread of transformed residuals across the range of predicted values, a uniform vertical spread indicating uniform variance.
- "Residuals vs. Leverage" highlights influential observations as those outside "Cook's distance lines," i.e., whose Cook's distance (the change in the predicted value if the given observation were omitted) is large.

The heteroscedasticity in residuals raises the concern that a subset of influential observations may have a disproportionate impact on the coefficients. The *Residuals vs. Leverage* plot shows residuals with high values (up to 15 standard deviations) but not high leverage.

S.3 Alternative multilevel specifications

S.3.1 Multilevel Gaussian model

To assess how the predictive performance changes when using a multilevel model, compared to the original specification, we fit, considering the original Gaussian functional form, the different multilevel models considered, namely: pooling regional intercepts only (model (4')) and pooling separately both regional intercepts and time periods (model (4")). Results are presented in Table S2. We find, as captured by the expected log predictive density (ELPD), that the partial pooling models perform marginally better than the model with fixed effects. However, they do not match the performance of the NB model which accounts for the count nature of the outcome data.

S.3.2 Pooling of period effects

Model (7) relaxes the assumption of unrelated effects across months by pooling the temporal effects, i.e., modeling them as random effects, and includes a time-varying linear climate predictor C_t that is experienced across all regions. This predictor for the country-wide period effect w_t is constructed from a relevant climate time series c_{it}, such as a drought index, or in our case, as it is readily available, the precipitation anomaly PA_{it}.

In our main specification, the climate predictor is defined as $C_t \equiv \sum_i \frac{c_{it}}{\mathbb{Q}_{c_i}(.5)}$, where $\mathbb{Q}_{c_i}(.5)$ is the median of the climate time series, so as to capture threshold exceedance. Alternatively, it could be defined so as to emphasize deviations from the average, such as $C_t^{\text{alt}} \equiv \sum_i \frac{c_{it}-\mu_{c_i}}{SD[c_i]}$, where μ_{c_i} is the mean of the climate time series and $SD[c_i]$ its standard deviation.

Figure S3 shows a graphical comparison of relevant statistics of the data observed—namely, the proportion of zeros, owing to the count nature of the outcome—against that of replicated datasets from the two model fits. The result suggests that the two models have comparable expected predictive ability. The estimation of their ELPD by leave-one-out cross-validation confirms this result, with a small and non-statistically significant difference of 1.2 in expected ELPD.

S.4 Estimation details

All estimations were conducted using the statistical software R (v4.5.1).

Single-level models. The linear regression for the replication exercise was estimated using the function `felm` of the `lfe` package (v3.1.1). The adjustment for serial and spatial autocorrelation, implemented in the original paper with the `ols_spatial_HAC` function available for Stata by Hsiang (2010)[53], was implemented by adapting the function `ConleySEs` available for R by Darin Christensen[54,55].

Residual plots were obtained with the R package `DHARMa` (v 0.4.7) which uses a simulation-based approach to create interpretable scaled residuals from generalized linear mixed models[56].

Bayesian models. All Bayesian models were estimated using the `rstanarm` package (v2.32.1), which interfaces with the Stan C++ library for Bayesian estimation. Each model was run with 4 chains, with 8000 iterations per chain (4000 warm-up). Convergence was assessed and ensured based on the potential scale reduction factor on split chains R-hat.

Each parameter was assigned the package's default "weakly informative" priors: centered autoscaled normal for slope coefficients and exponential(1) for the negative binomial dispersion parameter. These priors regularize estimation by ruling out extreme parameter values while leaving most likelihood information intact. Regression coefficients are given independent normal(0, 2.5) baseline priors, whose variances are then automatically adjusted to the scale of each predictor and outcome. Estimation is thereby primarily driven by the data and the partial pooling structure. The effective priors of the regressors in models 5 to 7 are presented in Table S3. For further details on the defaults, we refer the reader to the `rstanarm` documentation[57].

Because no external prior information was available, we chose weakly informative priors to minimize their influence. In such cases, the specific distributional form (e.g., normal or uniform) has little effect once the variance is set sufficiently large. More informative priors could be useful, especially with smaller or noisier datasets, if prior knowledge exists—for example, if posterior distributions from historical analyses were available. The Bayesian framework readily accommodates this flexibility, though such extensions are beyond the scope of the present study.

As estimation proceeds by simulation rather than analytic inversion of large matrices, numerical stability is not a concern. Convergence issues may arise if overly complex correlation structures were imposed in the priors.

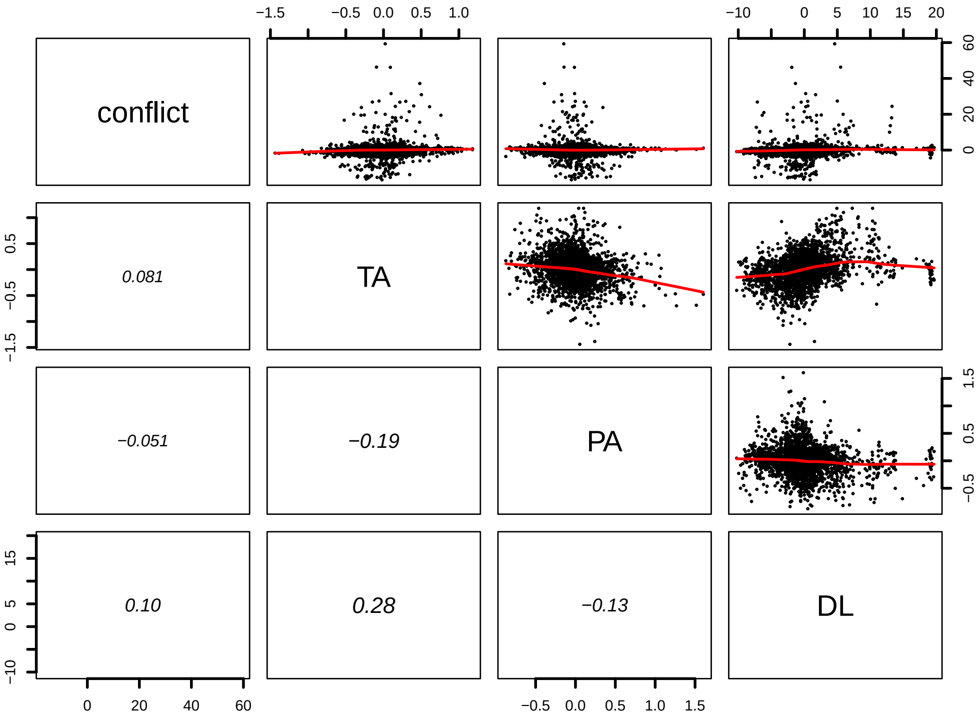

Figure S1. Scatterplots and correlations of the main variables after accounting for the region, month-year and region-month fixed effects. Top-panel: locally weighted scatterplot smoothing (LOWESS) lines with span=2/3.

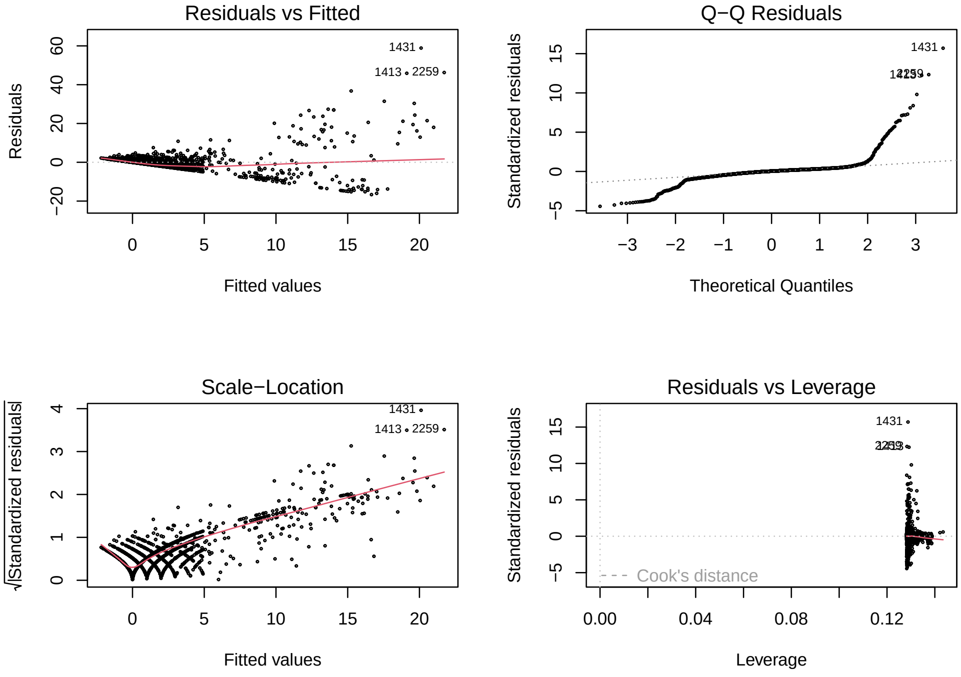

Figure S2. Diagnostic residual plots of the reduced-form model.

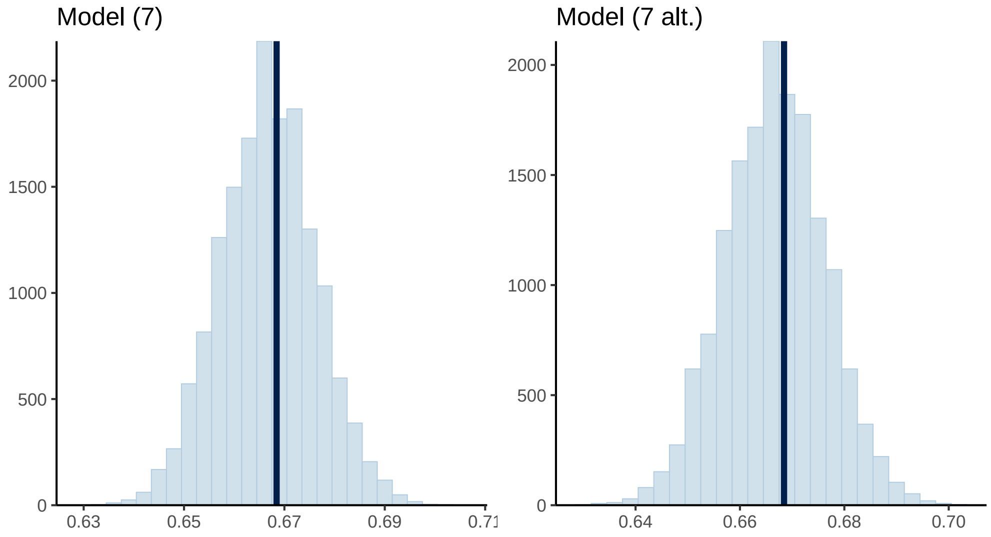

Notes: For each model, $S = 8000$ datasets are simulated from the posterior predictive distribution using the observed predictors. The light blue histogram represents the distribution of the value of the test statistic (the proportion of zeros) across the simulated datasets. The dark blue vertical line is the value of this statistic for the observed sample. The climate forcing $\overline{C}_{my}$ is defined as $\sum_i \frac{c_{imy}}{\mathbb{Q}_{c_i}(.5)}$ in model (7) and $\sum_i \frac{c_{imy} - \mu_{c_i}}{SD[c_i]}$ in model (7 alt.).

Figure S3. Graphical posterior predictive check: comparison of the proportion of zeros in observed vs simulated datasets; Models with partially-pooled period effects.

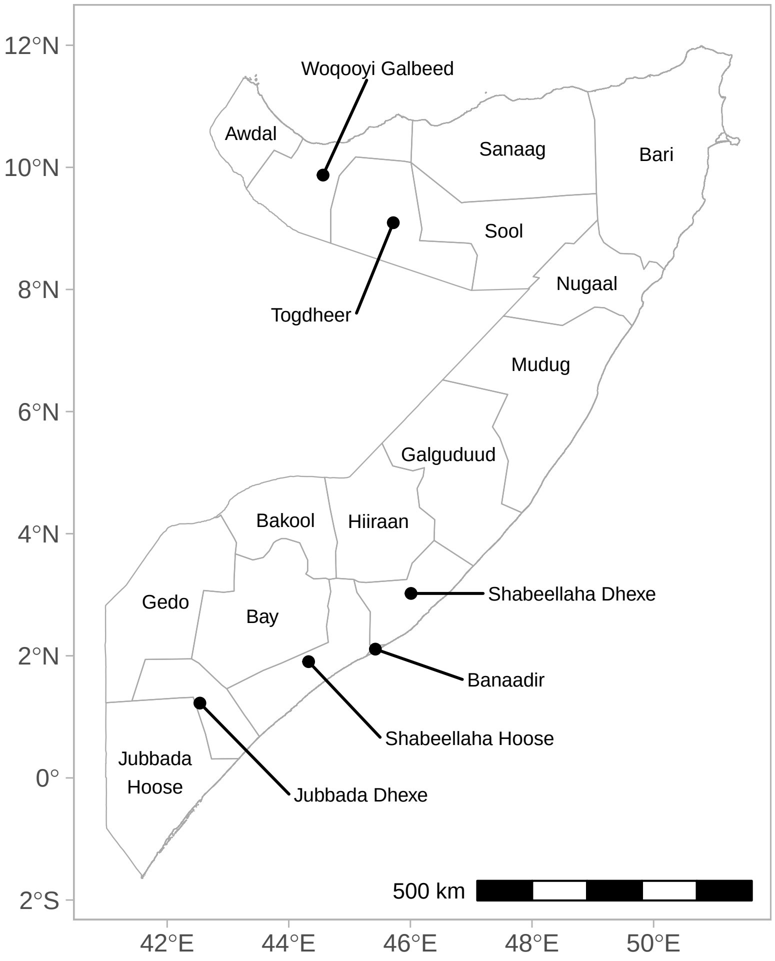

Figure S4. Map of the 18 administrative regions in Somalia. This figure was created using the statistical software R, version 4.5.1 (https://www.r-project.org).

	Backhaus et al. (2015)[18]	Berlemann and Tran (2020)[58]	Caruso et al. (2016)[59]	Hendrix and Salehyan (2012)[60]	Henry et al. (2004)[61]	Maystadt and Ecker (2014)[31]	Nawrotzki et al. (2013)[30]	Nawrotzki et al. (2015)[29]
**Pooling of individual effects**								
fully pooled (no FEs)			x	x	x			
no pooling (unit/group FEs)	x	x		x		x		
partial pooling (multilevel)							x	x
**Partial vs total effect of climate**								
reduced form w/o channels		x	x	x	x	x		
disentangle the reduced form relationship	x	x	x			x	x	x
**Functional form of the relationship with climate explanatory variables**								
linear	x	x	x	x	x	x	x	
non-linear				x			x	x
**Type of outcome variable**								
binary (event)				x	x		x	x
count or flow	x	x	x	x		x		
**Goal and use**								
association	x				x		x	x
prediction						x		
causal inference		x	x	x		x		
**Estimation**								
OLS, GLS	x	x	x		x	x		
ML		x		x			x	x
GMM								
Bayesian								
**Post-estimation standard error adjustment**								
Heteroskedasticity robust	x				x			
Clustering			x	x				
Serial/spatial correlation using a kernel						x		

Table S1. Attributes of regression-based approaches in the climate-fueled migration and conflict literatures, based on a sample of studies selected in systematic reviews.

Model	(4)	(4')	(4")	(5)	(6)	(7)
Condit. distribution	Normal	Normal	Normal	NB	NB	NB
TA	0.71	0.94	0.99	0.08	0.12	0.19
	(0.30)	(0.31)	(0.31)	(0.12)	(0.13)	(0.12)
DL	0.08	0.07	0.07	0.02	0.01	0.01
	(0.02)	(0.05)	(0.04)	(0.01)	(0.02)	(0.02)
PA	-0.47	-0.26	-0.21	-0.04	-0.03	-0.03
	(0.31)	(0.29)	(0.29)	(0.16)	(0.17)	(0.16)
$\overline{TA}_i$		-9.33	-7.73		-11.6	-11.13
		(2.53)	(2.57)		(2.37)	(2.23)
$\overline{DL}_i$		0.29	0.22		0.37	0.36
		(0.21)	(0.21)		(0.19)	(0.18)
$\overline{PA}_i$		-0.59	0.01		1.64	1.85
		(3.40)	(3.41)		(3.07)	(3.07)
$\overline{TA}_{my}$			-0.50			0.57
			(0.39)			(0.29)
$\overline{DL}_{my}$			-0.07			-0.07
			(0.04)			(0.04)
$\overline{C}_{my}$			0.01			0.01
			(0.01)			(0.00)
region	FEs	pooled	pooled	FEs	pooled	pooled
month	FEs	FEs	pooled	FEs	FEs	pooled
N	2,808	2,808	2,808	2,808	2,808	2,808
ELPD	-8090.2	-7815.7	-7787.3	-2861.7	-2833.8	-2809.8
$\text{ELPD}_{\text{diff}}$	-5280.3	-5005.9	-4977.5	-51.8	-24	0
$\text{SE}[\text{ELPD}_{\text{diff}}]$	186.4	204.5	213.3	13.8	9.5	0

Table S2. Bayesian central estimates of slope coefficients of single-level and multilevel models. For each regressor, the two sub-rows display summaries of its marginal posterior distribution: the distribution's median (top) and an estimate of the distribution's standard deviation (bottom, in parentheses), based on a scaling of the Median Absolute Deviation around that median. $\text{ELPD}_{\text{diff}}$ corresponds to the difference in ELPD between models, and $\text{SE}[\text{ELPD}_{\text{diff}}]$ to the standard error of that difference, where the reference is the model with the largest ELPD (model (7)).

Model(s)	Variable(s)	Regressor(s)	Distribution
(5), (6), (7)	α_0	TA	$\text{normal}(0, 4.21)$
(5), (6), (7)	γ_0	DL	$\text{normal}(0, 0.43)$
(5), (6), (7)	δ_0	PA	$\text{normal}(0, 5.76)$
(5)	$\{\phi_i\}$	$\{\mathbb{1}[region = i]\}$	$\text{normal}(0, 10.91)$
(5), (6)	$\{\psi_{my}\}$	$\{\mathbb{1}[year, month = my]\}$	$\text{normal}(0, 31.32)$
(6), (7)	η_1	$\overline{TA}_i$	$\text{normal}(0, 24.48)$
(6), (7)	η_2	$\overline{DL}_i$	$\text{normal}(0, 1.99)$
(6), (7)	η_3	$\overline{PA}_i$	$\text{normal}(0, 38.91)$
(7)	ψ_1	$\overline{TA}_{my}$	$\text{normal}(0, 4.9)$
(7)	ψ_2	$\overline{DL}_{my}$	$\text{normal}(0, 0.62)$
(7)	ψ_3	$\overline{C}_{my}$	$\text{normal}(0, 0.09)$
(5), (6), (7)	Θ		$\text{exponential}(1)$

Table S3. Prior distributions of the regression coefficients and reciprocal dispersion parameter of the multilevel negative binomial models.

References

1. Beine, M. & Jeusette, L. A meta-analysis of the literature on climate change and migration. *J. Demogr. Econ.* **87**, 293–344 (2021).
2. Berlemann, M. & Steinhardt, M. F. Climate change, natural disasters, and migration—a survey of the empirical evidence. *CESifo Econ Stud* **63**, 353–385 (2017).
3. Ide, T., Michael Link, P., Scheffran, J. & Schilling, J. The climate-conflict nexus: Pathways, regional links, and case studies. In Brauch, H. G., Oswald Spring, Ú., Grin, J. & Scheffran, J. (eds.) *Handbook on Sustainability Transition and Sustainable Peace*, 285–304 (Springer International Publishing, Cham, 2016).
4. Koubi, V. Climate change, the economy, and conflict. *Curr. Clim. Chang. Reports* **3**, 200–209 (2017).
5. Neumann, K. & Hilderink, H. Opportunities and challenges for investigating the environment-migration nexus. *Hum. Ecol. Interdiscip. J.* **43**, 309–322 (2015).
6. Sakaguchi, K., Varughese, A. & Auld, G. Climate wars? A systematic review of empirical analyses on the links between climate change and violent conflict. *Int. Stud. Rev.* **19**, 622–645 (2017).
7. Hermans, K. & Ide, T. Advancing research on climate change, conflict and migration. *DIE ERDE – J. Geogr. Soc. Berlin* **150**, 40–44, DOI: 10.12854/erde-2019-411 (2019).
8. Mach, K. J. *et al.* Climate as a risk factor for armed conflict. *Nature* **571**, 193–197, DOI: 10.1038/s41586-019-1300-6 (2019).
9. Cipollina, M., De Benedictis, L. & Scibè, E. Environmental migration? A systematic review and meta-analysis of the literature. *Rev. World Econ.* **160**, 1393–1441, DOI: 10.1007/s10290-024-00529-5 (2024).
10. Scartozzi, C. M. Reframing climate-induced socio-environmental conflicts: A systematic review. *Int. Stud. Rev.* **23**, 696–725, DOI: 10.1093/isr/viaa064 (2021).
11. Dell, M., Jones, B. F. & Olken, B. A. What do we learn from the weather? The new climate-economy literature. *J. Econ. Lit.* **52**, 740–798 (2014).
12. Burke, M., Hsiang, S. M. & Miguel, E. Climate and conflict. *Annu. Rev. Econ.* **7**, 577–617 (2015).
13. Carleton, T., Hsiang, S. M. & Burke, M. Conflict in a changing climate. *Eur. Phys. J. Spec. Top.* **225**, 489–511 (2016).
14. Hsiang, S. M. & Burke, M. Climate, conflict, and social stability: What does the evidence say? *Clim. Chang.* **123**, 39–55 (2014).
15. Gelman, A., Hill, J. & Vehtari, A. *Regression and Other Stories* (Cambridge University Press, 2020).
16. McLeman, R. Thresholds in climate migration. *Popul. Environ.* **39**, 319–338, DOI: 10.1007/s11111-017-0290-2 (2018).
17. Beine, M. & Parsons, C. Climatic factors as determinants of international migration. *Scand. J. Econ.* **117**, 723–767 (2015).
18. Backhaus, A., Martinez-Zarzoso, I. & Muris, C. Do climate variations explain bilateral migration? A gravity model analysis. *IZA J. Migr.* **4**, 3 (2015).
19. Conley, T. G. GMM estimation with cross sectional dependence. *J. Econom.* **92**, 1–45, DOI: 10.1016/s0304-4076(98)00084-0 (1999).
20. Newey, W. K. & West, K. D. A simple, positive semi-definite, heteroskedasticity and autocorrelation consistent covariance matrix. *Econometrica* **55**, 703–708 (1987).
21. Gelman, A. & Imbens, G. Why ask why: Forward causal inference and reverse causal questions. *NBER Work. Pap. 19614* (2013).
22. Shmueli, G. To explain or to predict? *Stat. Sci.* **25**, 289–310 (2010).
23. Rubin, D. B. Estimating causal effects of treatments in randomized and nonrandomized studies. *J. Educ. Psychol.* **66**, 688–701 (1974).
24. Ward, M. D., Greenhill, B. D. & Bakke, K. M. The perils of policy by p-value: Predicting civil conflicts. *J. Peace Res.* **47**, 363–375, DOI: 10.1177/0022343309356491 (2010).
25. Gelman, A., Meng, X.-L. & Stern, H. Posterior predictive assessment of model fitness via realized discrepancies (with discussion). *Stat. Sinica* **6**, 733–807 (2004).
26. Bell, A., Fairbrother, M. & Jones, K. Fixed and random effects models: making an informed choice. *Qual. & quantity* **53**, 1051–1074, DOI: 10.1007/s11135-018-0802-x (2019).

27. Wooldridge, J. M. *Introductory Econometrics: A Modern Approach* (South-Western Cengage Learning, 2012), 5th edn.

28. Bafumi, J. & Gelman, A. Fitting multilevel models when predictors and group effects correlate. *SSRN Electron. J.* DOI: 10.2139/ssrn.1010095 (2007).

29. Nawrotzki, R. J., Hunter, L. M., Runfola, D. M. & Riosmena, F. Climate change as migration driver from rural and urban Mexico. *Environ. Res. Lett.* **10** (2015).

30. Nawrotzki, R. J., Riosmena, F. & Hunter, L. M. Do rainfall deficits predict U.S.-bound migration from rural Mexico? Evidence from the Mexican Census. *Popul. Res. Policy Rev.* **32**, 129–158 (2013).

31. Maystadt, J.-F. & Ecker, O. Extreme weather and civil war: Does drought fuel conflict in Somalia through livestock price shocks? *Am. J. Agric. Econ.* **96**, 1157–1182 (2014).

32. von Uexkull, N. & Buhaug, H. Security implications of climate change: A decade of scientific progress. *J. Peace Res.* **58**, 3–17, DOI: 10.1177/0022343320984210 (2021).

33. Cohen, J. E., Roig, M., Reuman, D. C. & GoGwilt, C. International migration beyond gravity: A statistical model for use in population projections. *Proc. Natl. Acad. Sci.* **105**, 15269–15274 (2008).

34. Gelman, A. Causality and statistical learning. *Am. J. Sociol.* **117**, 955–966 (2011).

35. Armed Conflict Location and Event Data Project (ACLED) (2011).

36. University of East Anglia Climatic Research Unit. Climatic Research Unit (CRU): Time-series (TS) datasets of variations in climate with variations in other phenomena v3.1 (2008).

37. Hsiang, S. M. Standard error adjustment (OLS) for spatial correlation and serial correlation in panel data in Stata and Matlab. http://www.fight-entropy.com/2010/06/standard-error-adjustment-ols-for.html (2010). Accessed: 2021-11-21.

38. Almulhim, A. I. *et al.* Climate-induced migration in the global south: an in depth analysis. *npj Clim. Action* **3**, DOI: 10.1038/s44168-024-00133-1 (2024).

39. Cattaneo, C. *et al.* Human migration in the era of climate change. *Rev. Environ. Econ. Policy* **13**, 189–206 (2019).

40. Fussell, E., Hunter, L. M. & Gray, C. L. Measuring the environmental dimensions of human migration: The demographer's toolkit. *Glob. Environ. Chang.* **28**, 182–191 (2014).

41. Kaczan, D. J. & Orgill-Meyer, J. The impact of climate change on migration: A synthesis of recent empirical insights. *Clim. Chang.* **158**, 281–300 (2020).

42. Klaiber, H. A. Migration and household adaptation to climate: A review of empirical research. *Energy Econ.* **46**, 539–547 (2014).

43. McLeman, R. Developments in modelling of climate change-related migration. *Clim. Chang.* **117**, 599–611 (2013).

44. Rigaud, K. K. *et al.* *Groundswell: Preparing for Internal Climate Migration* (World Bank Group, 2018).

45. Zanhouo, D. A. K. & Nana, A. B. I. Modeling climate change impact on health and population migration: A systematic review. *Econ. Lit.* **1**, 51–65 (2019).

46. Sharifi, A. *et al.* Climate-induced stressors to peace: a review of recent literature. *Environ. Res. Lett.* **16**, 073006, DOI: 10.1088/1748-9326/abfc08 (2021).

47. Kniveton, D., Smith, C. & Wood, S. Agent-based model simulations of future changes in migration flows for Burkina Faso. *Glob. Environ. Chang.* **21**, S34–S40 (2011).

48. Hassani-Mahmooei, B. & Parris, B. W. Climate change and internal migration patterns in Bangladesh: an agent-based model. *Environ. Dev. Econ.* **17**, 763–780 (2012).

49. Ginnetti, J. Modelling displacement. *Forced Migr. Rev.* 10 (2015).

50. Barbieri, A. F. *et al.* Climate change and population migration in Brazil's northeast: Scenarios for 2025–2050. *Popul. Environ.* **31**, 344–370 (2010).

51. Krol, M. S. & Bronstert, A. Regional integrated modelling of climate change impacts on natural resources and resource usage in semi-arid Northeast Brazil. *Environ. Model. & Softw.* **22**, 259–268 (2007). Environmental Decision Support Systems.

52. Theisen, O. M., Gleditsch, N. P. & Buhaug, H. Is climate change a driver of armed conflict? *Clim. change* **117**, 613–625, DOI: 10.1007/s10584-012-0649-4 (2013).

53. Hsiang, S. M. Temperatures and cyclones strongly associated with economic production in the Caribbean and Central America. *Proc. Natl. Acad. Sci.* **107**, 15367–15372 (2010).

54. Christensen, D. Conley-SE function in Github (2017). Accessed: 2021-10-12.

55. Christensen, D. & Fetzer, T. Correcting for spatial and temporal auto-correlation in panel data (2015). Accessed: 2021-10-12.

56. Hartig, F. *DHARMa: Residual diagnostics for hierarchical (multi-Level / mixed) regression models* (2017). R package version 0.1.5. Accessed: 2021-11-21.

57. Gabry, Jonah and Goodrich, Ben and Stan Development Team. Prior distributions for rstanarm models. https://mc-stan.org/rstanarm/articles/priors.html (2023). Accessed: 2025-09-18.

58. Berlemann, M. & Tran, T. X. Climate-related hazards and internal migration empirical evidence for rural Vietnam. *Econ. Disasters Clim. Chang.* **4**, 385–409 (2020).

59. Caruso, R., Petrarca, I. & Ricciuti, R. Climate change, rice crops, and violence: Evidence from Indonesia. *J. Peace Res.* **53**, 66–83 (2016).

60. Hendrix, C. S. & Salehyan, I. Climate change, rainfall, and social conflict in Africa. *J. Peace Res.* **49**, 35–50 (2012).

61. Henry, S., Schoumaker, B. & Beauchemin, C. The impact of rainfall on the first out-migration: A multi-level event-history analysis in Burkina Faso. *Popul. Environ.* **25**, 423–460 (2004).
